# Propagule Pressure Build-Up by the Invasive *Hymenoscyphus fraxineus* Following Its Introduction to an Ash Forest Inhabited by the Native *Hymenoscyphus albidus*

**DOI:** 10.3389/fpls.2018.01087

**Published:** 2018-07-30

**Authors:** Ari M. Hietala, Isabella Børja, Halvor Solheim, Nina E. Nagy, Volkmar Timmermann

**Affiliations:** Department of Forest Health, Norwegian Institute of Bioeconomy Research, Ås, Norway

**Keywords:** ash dieback, climate, invasive species, population size, r/K selection

## Abstract

Dieback of European ash, caused by the ascomycete *Hymenoscyphus fraxineus* originating from Asia, has rapidly spread across Europe, and is threatening this keystone tree at a continental scale. High propagule pressure is characteristic to invasive species. Consistently, the enormous production of windborne ascospores by *H. fraxineus* in an ash forest with epidemic level of disease obviously facilitates its invasiveness and long distance spread. To understand the rate of build-up of propagule pressure by this pathogen following its local introduction, during 2011–2017 we monitored its sporulation at a newly infested ash stand in south-western Norway characterized with mild winters and cool summers. We also monitored the propagule pressure by *Hymenoscyphus albidus*, a non-pathogenic native species that competes for the same sporulation niche with *H. fraxineus*. During the monitoring period, crown condition of ash trees had impaired, and 20% of the dominant trees were severely damaged in 2017. *H. fraxineus* showed an exponential increase in spore production between 2012 and 2015, followed by drastic decline in 2016 and 2017. During 2011–2013, the two *Hymenoscyphus* species showed similar sporulation level, but thereafter spores of *H. albidus* were no longer detected. The data suggest that following local introduction, the population of *H. fraxineus* reaches rapidly an exponential growth stage if the local weather conditions are favorable for ascomata maturation across years. In the North Atlantic climate, summer temperatures critically influence the pathogen infection pressure, warm summers allowing the population to grow according to its biotic potential, whereas cold summers can cause a drastic decline in propagule pressure.

## Introduction

The invasive fungus *Hymenoscyphus fraxineus* (syn. *H. pseudoalbidus*, anamorph *Chalara fraxinea*) (Kowalski, 2006; Queloz et al., 2011; Baral et al., 2014) is threatening the future existence of European ash (*Fraxinus excelsior*) in Europe (Pautasso et al., 2013; Mitchell et al., 2014). This ascomycete has probably been introduced to Europe from Asia, where it is regarded as an endophyte with some parasitic capacity in leaves of Manchurian ash (*F. mandshurica*)(Drenkhan et al., 2017), a close relative of European ash. The dieback of European ash represents a showcase scenario of forest damage that can follow from exposure of evolutionary naïve trees to invasive alien pests that have a high level of adaptation to congeneric tree species in their native range. Since the first European record of ash dieback in Poland in the early 1990s (Przybył, 2002), the disease has spread across Central, Northern, Eastern and Western Europe (Timmermann et al., 2011; McKinney et al., 2014) and currently only populations at the southern and eastern range margins of European ash remain disease free (Solheim and Hietala, 2017).

In Europe, ash dieback has shown an annual spread rate of 50–75 km (Gross et al., 2014; Hamelin et al., 2016; Solheim and Hietala, 2017). The natural spread of *H. fraxineus* takes place by windborne ascospores released from ascomata formed on previous-year ash-leaf-litter. Typical to foliar pathogens, the ascospores of *H. fraxineus* are released during early morning during summer months (Timmermann et al., 2011; Hietala et al., 2013). While Steinböck (2013) and Chandelier et al. (2014) showed that the ascospore amount of *H. fraxineus* in air reaches a low plateau already a few hundred meters away from an infested stand, the effective dispersal distance of the pathogen propagules remains to be established. Besides ascospores, *H. fraxineus* produces also conidia that have been proposed to function as spermatia (review by Gross et al., 2014) or have a role in secondary local spread of the fungus (Fones et al., 2016). Particularly during the 1990s and early 2000s, when the causative agent of ash dieback still was unknown, trade and movement of infected ash seedlings (Sansford, 2013) and timber (Husson et al., 2012) may have facilitated the spread of ash dieback.

A study from south-eastern Norway located at the edge of continental climate, with average temperature around 15–16°C during the summer months (June–August), showed that *Hymenoscyphus fraxineus* exerts a high ascospore pressure in the period between mid-July and mid-August (Hietala et al., 2013). In the warmer climate of central Europe, a high pathogen propagule pressure can occur already in early June (Chandelier et al., 2014; Dvorak et al., 2016). The high propagule pressure obviously enables the fungus to overcome leaf defence responses and competition imposed by indigenous endophytes that are at this stage still in a quiescent growth mode characterized by low population density (Cross et al., 2017). There is increasing evidence that propagule pressure is a crucial ecological trait that influences the success of introduction but also the transition of invasive species to the subsequent stages; local establishment followed by spread outside the area of introduction and eventual widespread dominance (Lockwood et al., 2005; Colautti et al., 2006). The aim of the present study was to document the build-up rate of propagule pressure of *H. fraxineus* at an initially healthy ash stand. For the purpose, we installed a volumetric spore sampler in an ash stand at the west coast of Norway in 2011. In this region, the first signs of ash dieback were observed in some forests in 2010. At the experimental stand, we monitored the level of airborne *H. fraxineus* propagules over seven growing seasons. We also profiled the propagule level of *Hymenoscyphus albidus*, a fungus that competes for the same sporulation niche as *H. fraxineus*, but is indigenous to Europe and harmless to European ash.

## Materials and Methods

### Spore Sampling and Assessment of Tree Health Condition

To monitor the release of ascospores by *H. fraxineus* and *H. albidus*, in 2011 we installed a solar power-driven Burkard 7-day volumetric spore sampler (Burkard Scientific, Uxbridge, United Kingdom) at ground level in the center of a mixed deciduous stand dominated by European ash and located in Fana, 15 km south of Bergen (Bergen municipality, 60° 15′ 59″ N, 5° 20′ 12″ E, 30 m a.s.l.). The sampler was equipped with a standard wind vane that directs the trapping orifice into the wind. This stand was chosen because it was still devoid of ash dieback symptoms in 2011 and *H. albidus* ascomata were present at that time. At an air throughput of 10 l/min, airborne particles were collected on the adhesive-coated (50 ml vaseline, 6 g paraffin wax, toluene), transparent Melinex tape, fastened to a revolving drum which moved past the air intake of the spore sampler at a rate of 2 mm per hour. With the collection tape changed weekly, this provided a continuous temporal record of airborne propagules. In 2011, sampling started July 26th and was continued until September 5th. In the years 2012–2017 spore sampling was repeated, starting already during the second half of June and continuing until the beginning/mid of October, with the exception of 2016 when sampling was terminated September 19th. The spore sampler was kept precisely at the same position in the subjected stand across the seven monitoring seasons.

As reference, weather data were obtained from a meteorological station located at Bergen airport Flesland 7 km northwest of the studied ash stand ^1^. Missing data in some of measured meteorological parameters occurred due to technical problems at the station. Degree days were calculated by using a basal temperature of 5, 10, or 15°C.

In summer 2017, a damage assessment using defoliation as the main parameter (Eichhorn et al., 2016) was conducted on 20 dominant and 17 subdominant ash trees, subjectively selected and representative for the stand. Trees were considered to be healthy when they had up to 10% defoliation, slightly damaged when having 11–25% defoliation, moderately damaged when having 26–50% defoliation and severely damaged when the crown was more than 50% defoliated.

### DNA Isolation and Real-Time PCR

For two randomly selected days per each sampling week in each year, DNA was isolated from tape segments corresponding to the period from midnight to noon (24:00 – 12:00) with protocol #8 (Isolation of DNA from Mouse Tails) of Easy-DNA^TM^ Kit (Invitrogen, Carlsbad, CA, United States) according to the manufacturer’s instructions. The resulting 50-μl-volume DNA samples were stored at −20°C until processed by real-time PCR. As one spore sampler was employed, each sampling day represents a single biological replicate.

The real-time PCR detection of *H. fraxineus* and *H. albidus* DNA was performed using FAST BLUE qPCR MasterMix Plus w/o UNG for probe Assay Low ROX (Eurogentec, Seraing, Belgium) without any modifications in reagent concentrations. For detection of *H. fraxineus*, we used the forward primer Cfrax-F 5′-ATTATATTGTTGCTTTAGCAGGTC-3, reverse primer Cfrax-R 5′-TCCTCTAGCAGGCACAGTC-3′ and probe C-frax-P5′-FAM- CTCTGGGCGTCGGCCTCG-BHQ1-3′ designed and tested for species specificity by Ioos et al. (2009). For detection of *H. albidus*, we used the primer probe set designed and tested for species specificity by Husson et al. (2011), with the modification of using JOE as the reporter dye instead of YY: forward primer Halb-F 5′TATATTGTTGCTTTAGCAGGTCGC-3′, reverse primer Halb-R 5′-ATCCTCTAGCAGGCACGGTC-3′, and probe Halb-P5′-JOE-CCGGGGCGTTGGCCTCG-BHQ1-3′.

For quantification of *H. fraxineus* DNA, the primer and probe concentrations were 300 nM and 100 nM, respectively, as described by Ioos et al. (2009). For quantification of *H. albidus* DNA, 900 nM concentrations were used for the primers and the probe (Hietala et al., 2013).

To obtain fungal DNA for construction of standard curves, three Norwegian *H.* *fraxineus* (2009-106/1/2, 2009-107/1/2, 2009-112/1/3) and three *H. albidus* strains (2009-111/1/4, 2009-124/3/2, 2009-124/4/1), deposited at the culture collection of the Norwegian Institute of Bioeconomy Research, were grown for 3 weeks at 21°C on 2% malt extract agar coated by a cellophane membrane, and subjected to DNA isolation with a DNeasy plant mini kit (Qiagen) according to the manufacturer’s instructions. The obtained DNA was quantified by using micro-volume spectrophotometer NanoDrop 2000 (Thermo Scientific, Wilmington, DE, United States), and pooled together in equal concentrations to prepare fungal DNA standard curve samples that contained 10, 1, 0.1, 0.01, and 0.001 ng of *H. fraxineus* or *H. albidus* DNA in a volume of 3 μl. To ensure that the cycle threshold values from the experimental samples fell within the standard curves and to investigate the presence of compounds inhibitory to PCR, 3-log dilution series were prepared for all the experimental samples. Each experimental sample had undiluted DNA as the most concentrated, and all log dilutions of a sample were used as templates in real-time PCR. For both the experimental and standard curve samples, 3 μl of the DNA solution was used as the template for each 25-μl PCR reaction. Each reaction was repeated twice. PCR cycling parameters were 95°C for 10 min, followed by 40 cycles of 95°C for 15 s and 65°C for 55 s. Fluorescence emissions were detected with an ABI Prism 7700 (Applied Biosystems). The data acquisition and analysis were performed with the Sequence Detection System software package (1.7a; Applied Biosystems). Standard curves were constructed based on the relationship of *C*_t_ values and known DNA concentrations: the *C*_t_ values were plotted against log-transformed DNA amounts, and linear regression equations were calculated for the quantification of DNA pools by interpolation in unknown samples.

To obtain an estimate for ascospore amount of *H. fraxineus* and *H. albidus*, we divided their real-time PCR based DNA amount estimates by the weight of 0.0634 or 0.0549 pg that correspond to the estimated haploid genome sizes of *H. fraxineus* (62 Mb, McMullan et al., 2018) and *H. albidus* (53 MB, Sønstebø et al., 2017).

### Statistical Analyses

For each sampling year, Spearman’s rank correlation coefficients (ρ) were calculated for spore levels of *H. fraxineus* and *H. albidus* and weather parameters (mean daily temperature, precipitation, relative air humidity and wind speed), days with missing values either for the fungi or for the weather parameters were excluded from calculations. We also calculated the degree days for each year, by using 5, 10, or 15°C as basal temperatures. The relationship between initiation, peaking and ceasing of sporulation by *H. fraxineus* and degree days across the 7-year-long monitoring period was considered by calculating Pearson product moment correlations (*r*). ANOVA and Tukey HSD were used to consider species- and year-specific differences in maximum sporulation level; log transformation was made to reduce skewness in spore amount data. All the calculations were made by using SPSS 22.0 (IBM Inc., Armonk, NY, United States); correlations between variables and differences between treatments were considered significant at the 0.05 level.

## Results

### Development of Ash Health Condition

During installation of the spore sampler in 2011, no visible symptoms of ash dieback were detected in the stand and the health condition of the ash trees was generally considered as good, however, a structured assessment was not conducted. The results of the assessment in 2017 showed that 20% of the dominant trees were severely damaged, 35% moderately damaged, 35% slightly damaged and only 10% were still healthy (Supplementary Figure 1). Most of the severely damaged trees showed a large amount of epicormic shoots. Dead trees have so far not been observed in the stand.

### Changes in Infection Pressure by *H. albidus* and *H. fraxineus* During the Experimental Period

Spores of *H. albidus* were detected only during the first three seasons of monitoring, while spores of *H. fraxineus* were present in all seven sampling years. In 2011, 2012 and 2013, the mean spore levels of *H. albidus* during the 5 days with most spores detected (TOP_5_) were estimated as 217, 19 and 144, respectively. The respective TOP_5_ spore levels for *H. fraxineus* were 302 (2011), 120 (2012) and 1384 (2013), the levels in 2012 and 2013 being significantly higher than for *H. albidus*. After 2013, only spores of *H. fraxineus* were detected: the mean TOP_5_ spore levels were estimated as 35305 (2014), 437360 (2015), 20786 (2016) and 30 (2017). The increments in TOP_5_ spore level of *H. fraxineus* between 2013 and 2014, and between 2014 and 2015 were significant, as were the declines in top spore level between 2015 and 2016, and between 2016 and 2017. The exponential increase in maximum spore level between 2012 and 2015 can be described by the model *f*(x) = 6.7504e^2.7842x^ (*R*^2^ = 0.997), where x is the time in years (i.e., 1, 2012; 2, 2013 etc.).

### Within Season Variation in Sporulation Period and Peak by *H. albidus* and *H. fraxineus*

There was considerable variation between different years in the initiation, peaking and ceasing of sporulation by *H. albidus* and *H. fraxineus*. In 2011, when spore sampling was started first in late July and continued only to the beginning of September, the first detection, maximum level (a single day with most spores detected, TOP_1_) and last detection of *H. fraxineus* spores occurred on calendar days 209, 209 and 246, respectively, and those of *H. albidus* on calendar days 209, 209 and 239, respectively. Between 2012 and 2017, the spore sampling was started in the second half of June. In 2012 the first detection, TOP_1_ and last detection of *H. fraxineus* spores occurred on calendar days 204, 225 and 233, and those of *H. albidus* on calendar days 187, 197 and 211, respectively. In 2013 the first detection, TOP_1_ and last detection of *H. fraxineus* spores occurred on calendar days 200, 242 and 267, and those of *H. albidus* on calendar days 172, 176 and 249, respectively. From 2014 to 2017 spores of *H. albidus* were no longer detected, and the first detection, TOP_1_ and last detection of *H. fraxineus* spores occurred on calendar days 168, 201 and 276 (2014), 184, 240 and 272 (2015), 177, 225 and 260 (2016), 233, 236 and 236 (2017), respectively.

### Correlation of Weather and Sporulation of *H. albidus* and *H. fraxineus*

**FIGURE 1:**
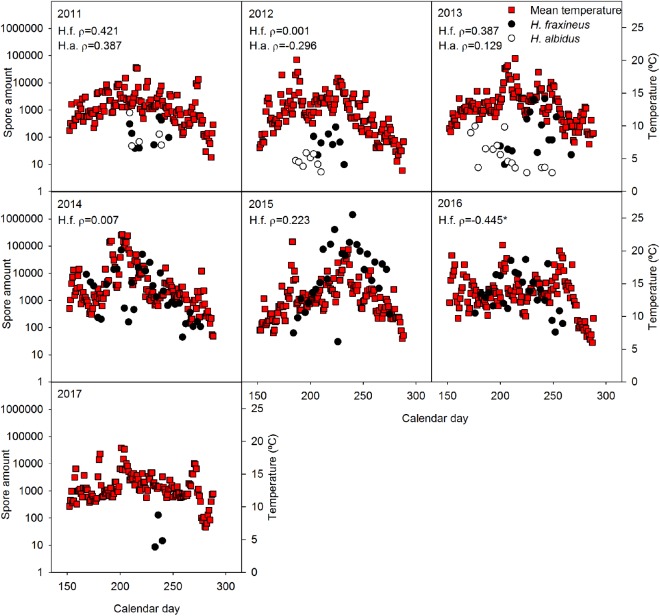
The amount of *H. fraxineus* (H.f.) and *H. albidus* (H.a.) spores 2011–2017 in relation to mean temperature (°C). The spore amount is calculated from amount DNA (pg/12 h) in the day period 12 midnight to 12 noon, each data-point constituting a single biological replicate. The spore data represent two randomly chosen days per each sampling week, whereas temperature data are provided for each day. Spearman correlation coefficient (ρ) between the spore amount and temperature data sets is enclosed, significant relationships being indicated by ^∗^.

**FIGURE 2:**
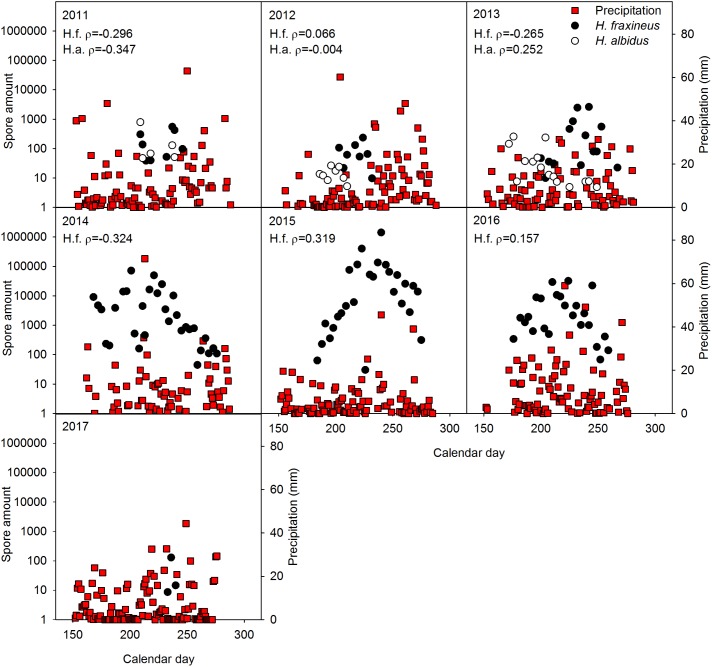
The amount of *H. fraxineus* (H.f.) and *H. albidus* (H.a.) spores during 2011–2017 in relation to precipitation (in mm). The spore amount is calculated from amount DNA (pg/12 h) in the day period 12 midnight to 12 noon, each data-point constituting a single biological replicate. The spore data represent two randomly chosen days per each sampling week, whereas precipitation data are provided for each day. Spearman correlation coefficient (ρ) between the spore amount and precipitation data sets is enclosed, significant relationships being indicated by ^∗^.

**FIGURE 3:**
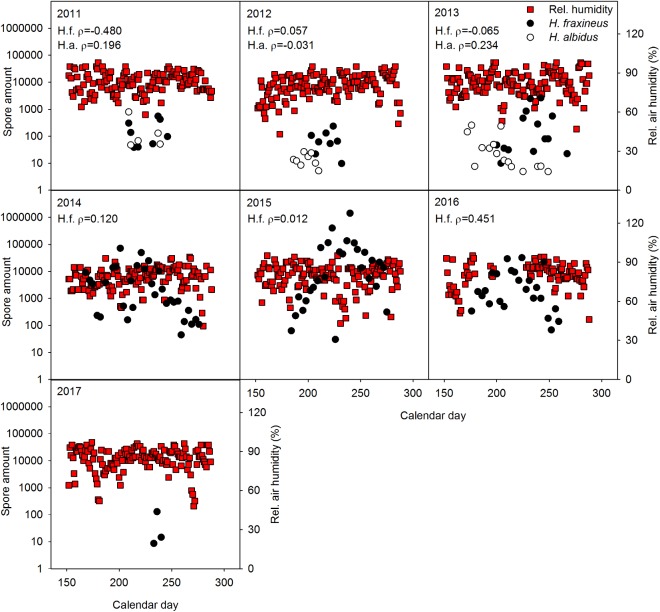
The amount of *H. fraxineus* (H.f.) and *H. albidus* (H.a.) spores during 2011–2017 in relation to relative air humidity (%). The spore amount is calculated from amount DNA (pg/12 h) in the day period 12 midnight to 12 noon, each data-point constituting a single biological replicate. The spore data represent two randomly chosen days per each sampling week, whereas air humidity data are provided for each day. Spearman correlation coefficient (ρ) between the spore amount and relative air humidity data sets is enclosed, significant relationships being indicated by ^∗^.

**FIGURE 4:**
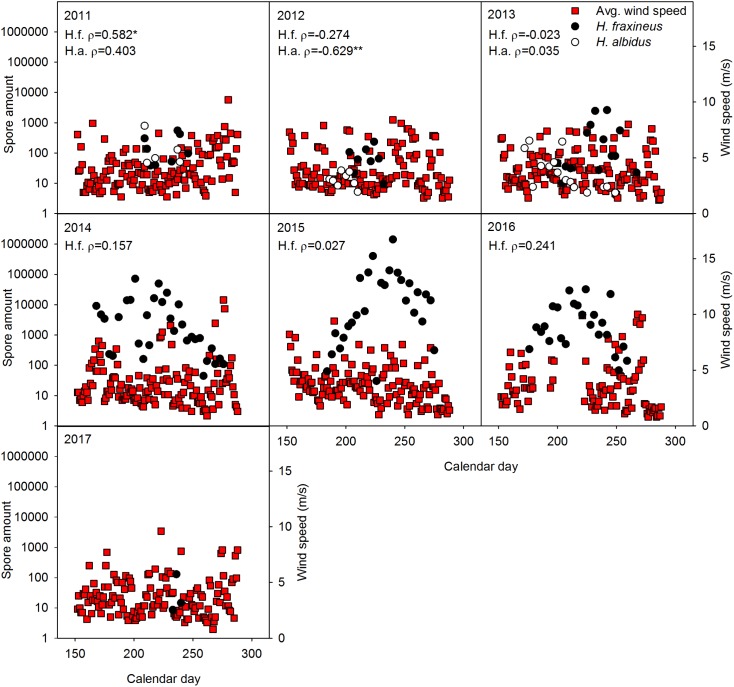
The amount of *H. fraxineus* (H.f.) and *H. albidus* (H.a.) spores during 2011–2017 in relation to average wind speed (m/s). The spore amount is calculated from amount DNA (pg/12 h) in the day period 12 midnight to 12 noon, each data-point constituting a single biological replicate. The spore data represent two randomly chosen days per each sampling week, whereas wind data is provided for each day. Spearman correlation coefficient (ρ) between the spore amount and average wind speed data sets is enclosed, significant relationships being indicated by ^∗^.

No significant relationship was generally observed between spore level of the two *Hymenoscyphus* species and temperature (**Figure 1**), precipitation (**Figure 2**), relative air humidity (**Figure 3**) or average wind speed (**Figure 4**) during the sporulation period. The exceptions were 2011 (ρ between *H. fraxineus* spore levels and average wind speed: 0.58), 2012 (ρ between *H. albidus* spore levels and average wind speed: −0.63) and 2016 (ρ between *H. fraxineus* spore levels and temperature: 0.45).

**FIGURE 5:**
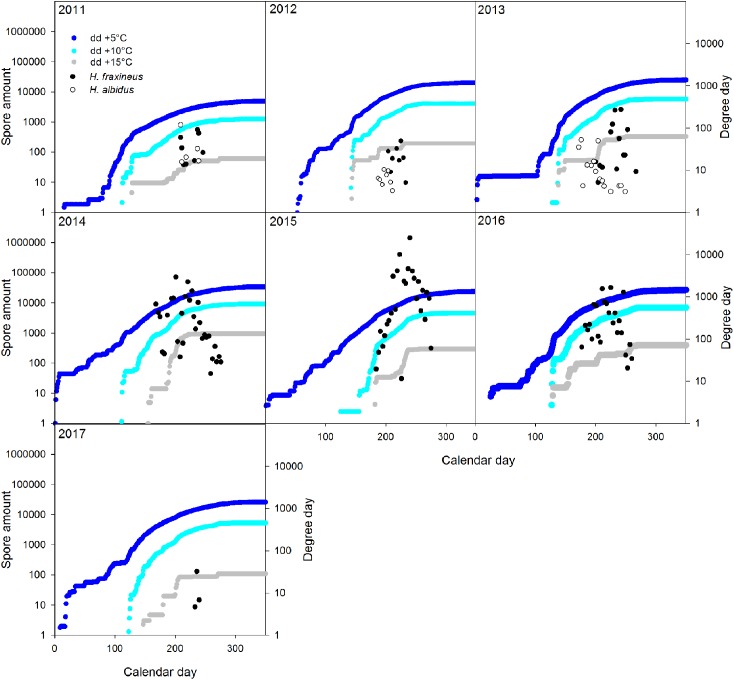
Accumulated degree days and amount of *H. fraxineus* and *H. albidus* spores detected 2011–2017. 5°C, 10°C or 15°C were used as basal temperature in degree day calculation. For the spores, each data-point constitutes a single biological replicate.

There were significant relationships between TOP_5_ spore level of *H. fraxineus* and the calendar day of first or last spore detection; the corresponding *r* were −0.85 and 0.86. We also calculated the relationship between fungal sporulation and degree days across the seven-year-long monitoring period, using 5°C (T_5_), 10°C (T_10_), or 15°C (T_15_) as basal temperatures (**Figure 5**). Since *H. albidus* was detected only between 2011 and 2013, this calculation was done only for *H. fraxineus*. The *r* between TOP_1_ spore level of *H. fraxineus* and sum of degree days by that calendar day were −0.29 (T_5_), −0.12 (T_10_) and 0.76 (T_15_), the latter being statistically significant. The *r* between the sum of degree days by August 31st and TOP_5_ spore level of *H. fraxineus* in the sampling year were 0.86 (T_5_), 0.87 (T_10_) and 0.82 (T_15_), all statistically significant. Since ascospore production in a given year is obviously influenced by the success of leaf infection during the previous year, we also calculated the relationship between the degree day sum by August 31st during the previous year and TOP_5_ spore level of *H. fraxineus* during the following year (i.e., degree day sum in 2010 vs. spore level in 2011); the corresponding *r* were 0.88 (T_5_), 0.90 (T_10_) and 0.92 (T_15_), all statistically significant.

## Discussion

The infection pressure by ascospores of *H. albidus* and *H. fraxineus* was comparable and low during the first year of monitoring. The noted level of *H. albidus* inoculum is consistent with the few records of its ascomata in Europe, the fungus being regarded as a relatively rare species (Baral and Bemmann, 2014). The lack of detection of *H. albidus* spores after 2013 suggests that the fungus was outcompeted by *H. fraxineus*, which showed an exponential increase in maximum spore level between 2012 and 2015. Studies from Denmark (Mckinney et al., 2012) and UK (King and Webber, 2016) also reported that *H. albidus* has become rare and probably even locally extinct due, whereas spore trapping studies from the Czech Republic, though based only on one year of sampling, imply that *H. albidus* could persist even after several years of competition by *H. fraxineus* (Dvorak et al., 2016; Koukol et al., 2016). For *H. albidus* detection, these Czech studies, like us, employed the primer/probe set designed by Husson et al. (2012). Whether the contrasting result could be influenced by study-specific differences in real-time PCR parameters (annealing temperature 65°C in the study of Husson et al. (2012) and the present work, and 60°C in the studies by Dvorak et al., 2016 and Koukol et al., 2016) is not clear to us. In Central Europe, first fruiting bodies of *H. fraxineus* appear already in May–June, distinctly earlier than *H. albidus*, and early initiation of sporulation has been proposed to provide an advantage to the invader (Baral and Bemmann, 2014). In the current stand sporulation of *H. fraxineus* initiated in 2012 and 2013 clearly later than that of *H. albidus*. This inconsistency between the present study and observations from central Europe relates presumably to different climatic conditions. Our ash stand represents oceanic climate with average daily temperatures normally between 13 and 14°C in summer, this being well below the optimum growth temperature that is close to 20–22°C for *H. fraxineus* (Kowalski and Bartnik, 2010; Pham et al., 2013) but also for *H. albidus* (Isabella Børja, pers. comm.). Taken together, the observations of fruiting body formation by *H. albidus* and *H. fraxineus* in central Europe and the sporulation data recorded now suggest that the order of sporulation initiation by *H. albidus* and *H. fraxineus* is not a decisive factor in their competition.

The exponential increase in maximum spore level of *H. fraxineus* between 2012 and 2015 is typical to invasive species, which may increase in population size according to their biotic potential in a new environment where access to resources is not a growth-limiting factor (e.g., Arim et al., 2006). Based on experiments under defined laboratory conditions, carpogenic germination of fungal pseudosclerotia to form fruiting bodies requires sufficient moisture, cumulative chill-hours and degree-days (e.g., Scherm et al., 2001). All these factors presumably influence also carpogenic germination of *H. fraxineus* pseudosclerotia but controlled experiments to determine the relations, to the best of our knowledge, have not yet been carried out. The region of the current stand is characterized by mild winters with average temperature around 1°C, high level of precipitation throughout the year and cool summers with average temperatures around 13–14°C in June, July and August. The significant correlation between annual maximum spore levels and degree-day sums suggest that in oceanic climate, cool summer temperatures are presumably the most limiting factor when it comes to carpogenic germination of *H. fraxineus* pseudosclerotia. An opposite scenario has been proposed for southern France, where high summer temperatures presumably restrict dissemination of *H. fraxineus* (Grosdidier et al., 2018). However, it is challenging to use field data to model the relationship between weather parameters and annual changes in species population size, especially during early stand infestation. At this phase invasive species may increase in population size according to their biotic potential (e.g., Arim et al., 2006), which makes distinguishing between inherent increase in population size and influence of weather on infection success and rate of carpogenic germination of pseudosclerotia difficult. For example, the maximum spore level of *H. fraxineus* was recorded in 2015, which was temperature-wise a typical summer in the region, with average temperature of 13.3°C in July which is usually the warmest month in the area. The previous summer was exceptionally warm, with average temperature of 17.8°C in July, and it seems plausible that besides the biotic potential of *H. fraxineus* to increase in population size, also the warm 2014 summer facilitated success of leaf infection and thereby contributed to the high level of spore production in 2015.

The drastic decline in sporulation level of *H. fraxineus* after 2015 resembles a population crash that takes place when a population overshoots the carrying capacity of the environment. Considering the increased defoliation of ash trees during the experimental period, the carrying capacity for *H. fraxineus* might have been reduced in the stand along with the degree of defoliation. Results from two of our ash dieback monitoring plots at the Norwegian west coast with a time series from 2012 have shown rapidly increasing damage and mortality for young and intermediate ash trees, while disease development in the large, dominant trees has been progressing slowly (Timmermann et al., 2017). The monitoring plots are located 110 km south and 40 km north, respectively, and show currently a health condition comparable to that observed in the present stand (Supplementary Figure 1). Regarding precipitation, rainfall can facilitate the deposition of spores on the ground and thereby reduce the amount of spores captured by volumetric air sampling, this wet deposition of spores becoming more important as the distance from spore source increases (Aylor and Sutton, 1992). The record level of precipitation in summer 2016 could have somewhat reduced aerial dispersal distances of *H. fraxineus* ascospores at the stand. However, the drastic reduction in spore levels of *H. fraxineus* between 2016 and 2017 can hardly be explained by changes in the carrying capacity alone or by rainfall scavenging of spores – it is likely that the low degree-day sum in June and July in 2016 and especially in 2017 were less favorable to carpogenic germination of *H. fraxineus* pseudosclerotia. In this context it is highly significant that *H. fraxineus* can survive several months in air-dried petioles covered with a pseudosclerotial layer (Gross and Holdenrieder, 2013). Further, ascomata of *H. fraxineus* can be formed not only in the year after leaf fall but also on older petioles, at least up to five growing seasons after the leaves have been shed (Kirisits, 2015). This testifies for a strong competence in defending the saprobic niche against abiotic and biotic stress, an ability that may allow survival of *H. fraxineus* through unfavorable seasons for ascomata production. Should the summer 2018 turn out to be warm in southwestern Norway, we predict that the spore levels of *H. fraxineus* increase again in the subjected stand, via carpogenic germination of pseudosclerotia formed during previous years.

According to the stochastic niche theory of resource competition, invasion and community assembly (Tilman, 2004), the probability that an invader can survive, reach maturity and reproduce depends on its traits relative to the traits of the established species. This theory predicts that successful invaders should decrease the abundance mainly of species that are competitively similar to themselves. The genome sequence data indicate that *H. albidus* and *H. fraxineus* are very close to each other on the trade-off surface (Stenlid et al., 2017) but the species show some obvious differences in life history traits (e.g., review by Hietala et al., 2018). The largest ascomata recorded for *H. fraxineus* and *H. albidus* were 8.5 and 4 mm in diameter, respectively (Baral and Bemmann, 2014). Due to the larger hymenium, *H. fraxineus* has probably a higher ascospore production than *H. albidus*. Moreover, in laboratory experiments *H. fraxineus* shows a considerably larger ascocarp production capacity than *H. albidus* (Wey et al., 2016), and in forest *H. fraxineus* can form as many as 10000 ascocarps per m^2^ (Hietala et al., 2013) or 13 million per hectare (Kowalski et al., 2013). As discussed by Wey et al. (2016), *H. albidus* may simply have inherently low fecundity, and thereby be at a disadvantage to *H. fraxineus*. When it comes to adaptation to changes in environmental conditions, a characteristic of invasive species, also in offspring quality *H. fraxineus* has a clear advantage over *H. albidus*; while *H. fraxineus* is an outcrossing heterothallic species with recombinant offspring, *H. albidus* is homothallic species reproducing via haploid selfing (Gross et al., 2012; Wey et al., 2016; Brasier et al., 2017).

Besides offspring quantity and quality, *H. fraxineus* differs from *H. albidus* owing to its extensive pseudosclerotial layers that may cover the entire vein system of the compound leaf, while the pseudosclerotial layer of *H. albidus* appears usually sharply isolated on the otherwise straw-colored rachis (Baral and Bemmann, 2014). The high investment in survival structures, high fecundity and production of recombinant offspring by *H. fraxineus* may represent an adaptation to harsh environmental conditions, the native hosts of the fungus spanning across multiple climate zones in Asia, these including regions with cold dry winters and either hot, warm or cold summers (Wei et al., 2004; Peel et al., 2007). To our knowledge, there are no other long-term data available about annual changes in *H. fraxineus* population size, either from Asia or from Europe. It is noteworthy that in European arboreta the Asian host trees of *H. fraxineus* support equally well pathogen ascomata formation as common ash (Nielsen et al., 2017). We hypothesize that under Asian climatic conditions *H. fraxineus* is not able to grow according to its biotic potential over several years and rarely reaches a critical population size when crowding effects become a selective force, whereas the more stable continental climate of Europe enables *H. fraxineus* to frequently increase in population size over several years, to the effect that the fungus overshoots the environment carrying capacity and causes ash decline. We predict that the boom and bust cycle now observed in *H. fraxineus* sporulation level is not specific to the subjected stand but a more general phenomenon, and that the length of the exponential increase in *H. fraxineus* population size depends on how stable the climatic conditions that favor ascocarp sporulation and leaf infection are locally across consequent years. Our results are coherent with the predictions of Goberville et al. (2016), who proposed that climate change will expand the range of *H. fraxineus*.

## Author Contributions

AH, IB, HS, NN, and VT contributed to the design of the project, analysis and interpretation of data, and to drafting and revising of the manuscript. Processing of qPCR data was done by AH. AH and NN conducted the statistical analysis.

## Conflict of Interest Statement

The authors declare that the research was conducted in the absence of any commercial or financial relationships that could be construed as a potential conflict of interest.

## Footnotes

1 eklima.met.no
