# Supplementary material for: Propagule Pressure Build-Up by the Invasive Hymenoscyphus fraxineus Following Its Introduction to an Ash Forest Inhabited by the Native Hymenoscyphus albidus
Source: Front Plant Sci. 2018 Jul 30;9:1087. doi: 10.3389/fpls.2018.01087 (PMC6077690; doi:10.3389/fpls.2018.01087)
Supplement: Supplementary file 1 [file Image_1.PDF]

Supplementary Material

Propagule pressure build-up by the invasive *Hymenoscyphus fraxineus* following its introduction to an ash forest inhabited by the native *Hymenoscyphus albidus*

Ari M. Hietala*, Isabella Børja, Halvor Solheim, Nina E. Nagy, Volkmar Timmermann

*** Correspondence:** Ari M. Hietala ari.hietala@nibio.no

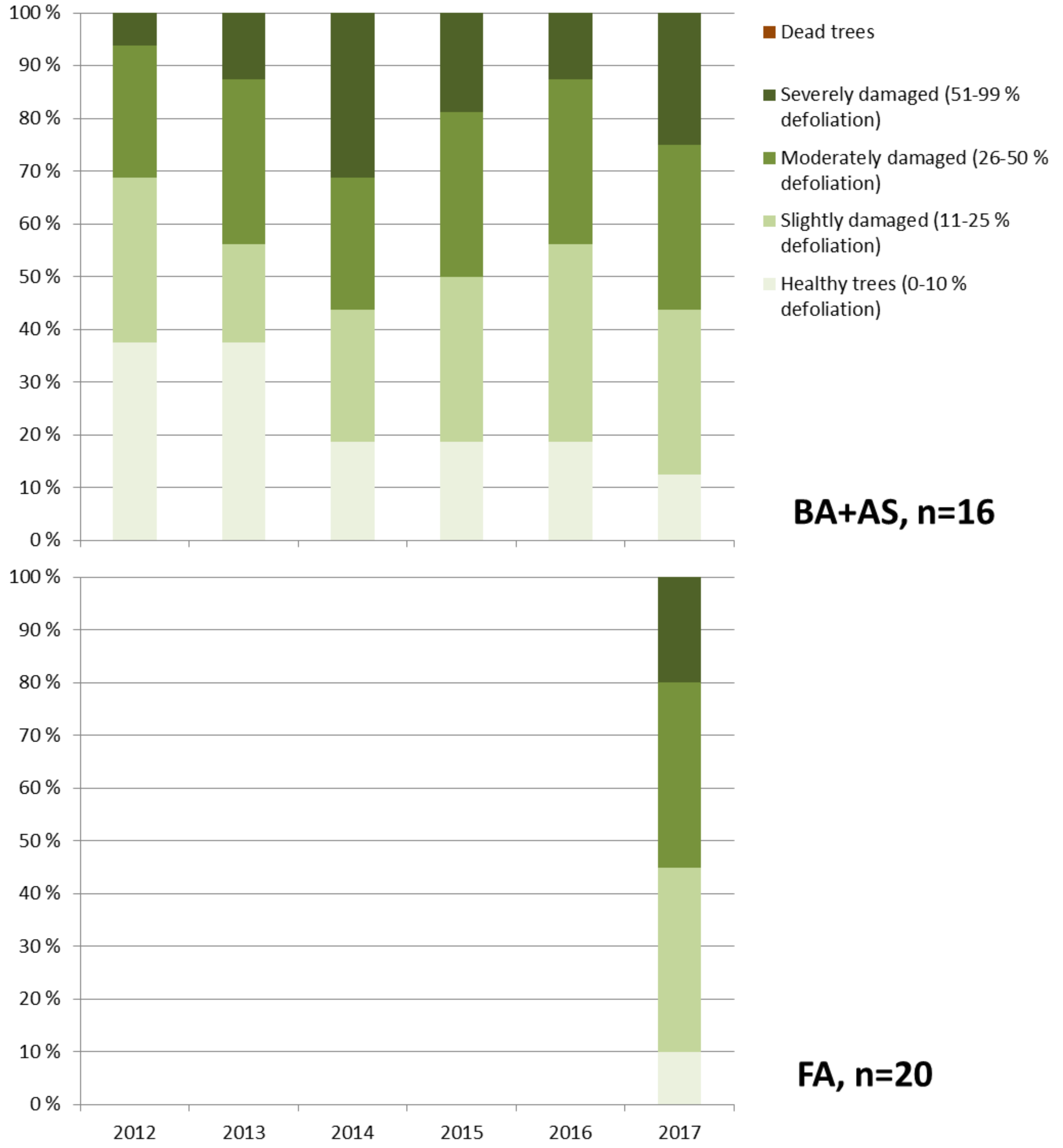

Supplementary Figure 1. Percentage of dominant ash trees (DBH>125 mm) in different damage classes. Upper panel: Monitoring plots Baustad (BA) and Askvik (AS) in Western Norway, damage assessments 2012–2017. Lower panel: Experimental stand in Fana, Bergen (FA), damage assessment 2017.
